# Cell-type-specific translational control of spatial working memory by the cap-binding protein 4EHP

**DOI:** 10.1186/s13041-023-00995-2

**Published:** 2023-01-18

**Authors:** Shane Wiebe, Ziying Huang, Reese Jalal Ladak, Agnieszka Skalecka, Roberta Cagnetta, Jean-Claude Lacaille, Argel Aguilar-Valles, Nahum Sonenberg

**Affiliations:** 1 Department of Biochemistry, McGill University, McIntyre Medical Building, 3655 Promenade Sir William Osler, Montreal, QC H3G 1Y6 Canada; 2 Goodman Cancer Institute, 1160 Pine Avenue West, Room 614, Montreal, QC H3A 1A3 Canada; 3 Department of Neuroscience and CIRCA, University of Montreal, Succ. Downtown, P. O. Box 6128, Montreal, QC H3C 3J7 Canada; 4 Department of Neuroscience, Carleton University, Health Sciences Building, 1125 Colonel By Drive, Ottawa, ON K1S 5B6 Canada

**Keywords:** Glutamatergic neurons, GABAergic neurons, eIF4E homologous protein (4EHP), Mechanistic target of rapamycin complex 1 (mTORC1)

## Abstract

The consolidation of learned information into long-lasting memories requires the strengthening of synaptic connections through de novo protein synthesis. Translation initiation factors play a cardinal role in gating the production of new proteins thereby regulating memory formation. Both positive and negative regulators of translation play a critical role in learning and memory consolidation. The eukaryotic initiation factor 4E (eIF4E) homologous protein (4EHP, encoded by the gene *Eif4e2*) is a pivotal negative regulator of translation but its role in learning and memory is unknown. To address this gap in knowledge, we generated excitatory (glutamatergic: CaMKIIα-positive) and inhibitory (GABAergic: GAD65-positive) conditional knockout mice for 4EHP, which were analyzed in various behavioral memory tasks. Knockout of 4EHP in *Camk2a*-expressing neurons (4EHP-cKO^exc^) did not impact long-term memory in either contextual fear conditioning or Morris water maze tasks. Similarly, long-term contextual fear memory was not altered in *Gad2*-directed 4EHP knockout mice (4EHP-cKO^inh^). However, when subjected to a short-term T-maze working memory task, both mouse models exhibited impaired cognition. We therefore tested the hypothesis that de novo protein synthesis plays a direct role in working memory. We discovered that phosphorylation of ribosomal protein S6, a measure of mTORC1 activity, is dramatically reduced in the CA1 hippocampus of 4EHP-cKO^exc^ mice. Consistently, genetic reduction of mTORC1 activity in either excitatory or inhibitory neurons was sufficient to impair working memory. Taken together, these findings indicate that translational control by 4EHP and mTORC1 in both excitatory and inhibitory neurons are necessary for working memory.

**Supplementary Information:**

The online version contains supplementary material available at 10.1186/s13041-023-00995-2.

## Introduction

Experiments in the 1960’s provided the first evidence for an essential role of protein synthesis in memory formation [1]. Mice trained in a Y-maze shock avoidance task were unable to recall which arm of the Y-maze was linked to the aversive shock 3 days after bilateral intracerebral injection of the protein synthesis inhibitor, puromycin [1]. However, the use of protein synthesis inhibitors provides only limited insight into the biomolecular processes underlying memory. Genetic animal models served as essential tools towards this end. One of the seminal discoveries first documenting that translational control mechanisms govern memories showed that preventing the phosphorylation of eukaryotic initiation factor (eIF)2α, a molecular brake mechanism for protein synthesis, increased general neural translation and enhanced long-term memory (LTM) [2, 3].

mRNA translation is highly regulated at the initiation stage by a heterotrimer protein complex termed eIF4F which interacts directly with the mRNA 5’ cap structure (m7GpppN cap, where N is any nucleotide and m is a methyl group). eIF4F is composed of a cap-binding protein eIF4E, a molecular scaffolding protein eIF4G, and an mRNA helicase eIF4A [4]. Disrupting the interaction between eIF4E and eIF4G using the inhibitor 4EGI-1 prevents LTM [5]. The eIF4E homologous protein (4EHP or eIF4E2) is also a 5’ cap-binding protein, but in contrast to eIF4E it cannot interact with eIF4G and represses translation initiation under most circumstances [6] (hypoxia being an exception [7]). 4EHP inhibits translation via the microRNA (miRNA)-induced silencing complex (miRISC) [8, 9]. Despite having a weaker cap-binding affinity than eIF4E [10], 4EHP can compete with eIF4E for binding the cap upon recruitment by the 4E transporter protein (4E-T) and miRISC [8]. Importantly, translational control via miRNA-induced mRNA silencing plays a critical role in synaptic plasticity and memory [11]. Previously, we identified a critical role for 4EHP in regulating synaptic plasticity (long-term depression) and mediating social behaviors [12].

Since 4EHP is important for brain function, most likely through miRNA regulation of specific mRNAs important for plasticity-related events at the synapse, we investigated its cell-type-specific activity in learning and memory. To this end, we generated 4EHP conditional knockout (cKO) mice using the Cre-Lox system in excitatory and inhibitory neurons. We did not observe alterations in LTM, but rather an impairment in short-term working memory (WM). We observed reduced ribosomal protein S6 phosphorylation, a prominent readout of the mechanistic target of rapamycin complex 1 (mTORC1) activity, in CA1 hippocampal pyramidal neurons of 4EHP-cKO^exc^ mice. Consistently, conditional deletion of Raptor, the defining component of mTORC1, resulted in impaired WM. Deletion of 4EHP in GABAergic neurons likewise resulted in impaired WM without affecting LTM. The latter effect was likely due to a developmental abnormality, since the total number of GAD67-positive cells was reduced.

Taken together, our findings reveal an important cell-type-specific role of 4EHP in regulating WM possibly through direct modulation of mTORC1 activity, consistent with the known and well-established role of miRNA-mediated regulation of the mTORC1 signaling pathway [13–17], including in the brain [18].

## Materials and methods

### Mice

Adult (i.e. postnatal day [P] 60–90 [19]) male mice on Jackson Laboratory C57BL/6J background were used for experiments. Mice were housed by sex and genotype after weaned at P21 in groups of 2–5 animals per cage. Mice were kept at standard room temperature (RT): 20–22 °C on a 12 h light/dark cycle (7:00–19:00 light period) with food and water access ad libitum. Behavioral experiments were conducted in a soundproof room between 8:00 and 16:00. All behavioral apparatuses were cleaned between animals. Mice were handled 2 times (once per day for 2 days) and habituated in the behavioral room for 20 min prior to behavioral testing. See below for detailed behavioral methods. For data acquisition, analysis and manual scoring, the experimenter was blind to mouse genotype which was randomized throughout the day and across days (in the case of multi-day experiments). Animal care, handling, and experiments were performed according to the guidelines of the Canadian Council on Animal Care and approved by the McGill University Animal Care Committee.

### Generating conditional knockout (KO) mice

To conditionally delete 4EHP in excitatory neurons, we crossed *Eif4e2*^*flx/flx*^ mice [20] with CaMKIIα-Cre mice (glutamatergic forebrain neurons where Cre recombinase activity has been reported to occur at postnatal day (P) 19 [21], JAX stock no. 005359, on C57BL/6 background). *Eif4e2*^+*/flx*^*:Camk2a-Cre* mice were used to breed F2: *Eif4e2*^+*/*+^*:Camk2a-Cre* (referred to in the text as 4EHP-WT) and *Eif4e2*^*flx/flx*^*:Camk2a-Cre* (referred to in the text as 4EHP-cKO^exc^). F3 mice were used for experiments and housed according to genotype. The same breeding scheme was used to generate *Eif4e2*^+*/*+^*:Gad2-Cre* (referred to in the text as 4EHP-WT) and *Eif4e2*^*flx/flx*^*:Gad2-Cre* (referred to in the text as 4EHP-cKO^inh^) using GAD65-Cre mice (GABAergic interneurons, where Cre recombinase activity occurs around embryonic day (e) 15 [22], JAX stock no. 010802, on C57BL/6 background). *Rptor*^*flx/flx*^*:Camk2a-Cre* and *Rptor*^*flx/flx*^*:Gad2-Cre* mice were used as previously characterized [23]. Comparisons were made between + */* + and *flx/flx* mice expressing Cre to normalize for any confounding effects generated by the presence of Cre recombinase alone.

### Genotyping

Mouse genotype was determined for each animal using PCR and gel electrophoresis. *Eif4e2* gene was amplified using 5’-TCAGAGCAAGAACACTTACAGGACCAAG forward and 5’-GGCCCAGCCTGCCTGGCATTCTAGTGG reverse primers. The PCR product was separated through a 1.5% agarose gel using a 150 V potential difference. WT bands were detected around 700 bp and floxed bands at 850 bp. To detect the presence of Cre (300 bp), the forward 5’-GATTGCTTATAACACCCTGTTACG and reverse 5’-GTAAATCAATCGATGAGTTGCTTCA primers were used.

### Western blotting

Soluble protein lysates were prepared by homogenizing brain tissue (from 7 to 9 mice, depending on the experiment) using a pestle grinder in radioimmunoprecipitation assay (RIPA) buffer (R0278, Sigma) on ice containing proteinase (05892970001, Roche) and phosphatase inhibitors (P5726 and P0044, Sigma). Samples were first incubated on ice for 30 min then centrifuged at 16,000 *g* for 20 min at 4 °C. 25 µg of protein from the supernatant were loaded onto a polyacrylamide gel (final concentration: 12% Acrylamide/Bis Solution, 29:1, 375 mM Tris–HCl pH 8.8, 0.1% SDS, 0.1% TEMED, and 0.1% ammonium persulfate) and separated using a potential difference of 100 V. Protein was then transferred onto a nitrocellulose membrane at 25 V overnight at 4 °C in transfer buffer (25 mM Tris–HCl pH 8.3, 190 mM glycine, and 20% methanol). Membranes were then incubated with 5% bovine serum albumin (BSA) in Tris-Buffered Saline with Tween 20 (TBST, 20 mM Tris–HCl pH 7.5, 150 mM NaCl, 0.1% Tween 20) for 1–2 h at RT to reduce non-specific binding. Membranes were then probed with one of the following primary antibodies at the indicated dilution: EIF4E2 (GTX103977, GeneTex, 1:500), GAPDH (ab9482, Abcam, 1:40 000), diluted in TBST with 5% BSA overnight at 4 °C (or 1 h at RT for GAPDH). Secondary antibody conjugated to horseradish peroxidase (HRP, anti-mouse and anti-rabbit, GE Healthcare) was diluted 1:5000 in TBST with 5% BSA and added to membranes for 1–2 h at RT. Membranes exposed to Blu-Lite UHC Ultra-High Contrast Western blotting film (A8815, MTC Bio) after incubating in enhanced chemiluminescence (Western Lighting® Plus ECL, 0RT2655:0RT2755, Perkin Elmer) for 1 min. Quantification of the band intensity was done using Image J software (NIH).

### Immunofluorescence on brain slices

Mice were placed under general isoflurane anesthesia until loss of pain reflex. Mice were then transcardially perfused with filtered ice-cold PBS then ice-cold 4% PFA. Dissected brains were placed in ice-cold 4% PFA overnight at 4 °C for post-fixation and then moved to 30% sucrose in PBS for 3 d at 4 °C for cryoprotection. 20 µm coronal sections were prepared using a cryostat and adhered to glass coverslips (12–550-15, Fisher). Sections were placed in boiling 10 mM sodium citrate buffer, pH 6.0 for 20 min for antigen retrieval after being washed 3 times in PBS for 5 min. Sections were placed in blocking solution (10% BSA and 0.5% Tween 20 in PBS) for 1–2 h at RT. The following primary antibodies were used to probe for: eIF4E2 (sc-100731, Santa Cruz), CaMKIIα (sc-13141), GAD67 (ab213508, Abcam), Phospho-S6 Ribosomal Protein (Ser240/244) (D68F8) XP (5364, Cell Signaling Technology), EMX1 (PA5-35373, Thermo), PVALB (195004, Synaptic System), Somatostatin 28 (ab111912, Abcam), Laminin (L9393, Sigma) diluted 1:100 in blocking solution overnight at 4 °C. Sections were incubated with Alexa-conjugated secondary antibodies (1:300) and Hoechst (1:1000) diluted in blocking buffer for 1–2 h at RT in the dark. Coverslips were mounted with DAKO. Samples were visualized 24 h later with a ZEISS Laser Scanning Microscope 880. Images were quantified using Image-J software. When quantifying fluorescence intensity in inhibitory neurons, the Mean Gray Value (MGV) was scored since this normalizes the area of each cell. For all other quantification, the Integrated Density was scored.

### Morris water maze

The Morris water maze (MWM) memory task was performed as previously described [24]. Mice were trained to locate a hidden platform with either 1 trial per day, maximum 60 s per trial (weak) or 3 trials per day, maximum 120 s per trial (strong) over 5 consecutive days in a circular pool 1 m in diameter. Learning was determined manually by timing the latency to locate the hidden (submerged) platform (i.e. escape latency). For probe trials on the following day (day 6), the platform was removed from the maze and the animals were given 60 s to navigate the maze. The percentage of time spent in each quadrant of the maze (quadrant occupancy) was recorded using an automated video tracking system (HVS Image, Buckingham, UK).

### T-maze

The T-maze is a test to measure spatial WM. The T-maze consists of a runway (stem) with a left and right arm choice at the end. The maze is enclosed by short walls so the animal can navigate using cues in the environment. Mice were individually placed at the bottom of the stem. For the training phase, one of the maze arms was closed and the animal was allowed to freely explore the other maze arm for 10 min. After 1 h, the mouse was reintroduced to the maze with exception that the animal could enter the previously closed and unexplored arm. We used the animal’s innate preference for novelty to probe their ability to alternate exploratory behavior of the previously unencountered T-maze arm. Each trial was recorded using an overhead camera and performance was quantified manually. Time spent in the familiar arm and novel arm were scored. From this data we further calculated discrimination index (DI, see equation below).

$$DI(\%)=\frac{novel\,arm-familiar\,arm}{total\,exploration\,time}\times 100$$

### Novel object recognition

We assessed working memory using a short-term object recognition test. Mice were first placed inside a white box with an open top measuring 50 × 50 × 30 cm. On day 1, mice were habituated to the empty box once for 10 min. After 24 h, mice were placed back in the box with two identical objects and allowed to explore them for 10 min (training). After 1 h, mice were placed back in the box where one of the two objects were replaced with a novel object. Mice freely explored the objects for 10 min (test). Time spent exploring the familiar object and novel object were scored. From this data we derived the discrimination index (DI, see equation above).

### Contextual fear conditioning

Mice were placed in a sound-proof box containing an enclosed isolation chamber with an electric grid floor and overhead camera. Mice were recorded for 2 min before receiving a mild foot shock (0.7 mA, 1 s). After 1 min, mice were removed and placed back in their home cage. 1 or 24 h later, mice were placed back in the enclosure (context) and recorded for 4 min. The average percent freezing over 4 min was used as an assessment of LTM.

### Statistical analysis

Statistical analysis was performed on GraphPad Prism 9. An unpaired t-test was used to compare one experimental parameter. Mixed design two-way ANOVA was used to compare two experimental parameters (i.e. genotype as an independent variable and arms in the elevated plus maze test as a repeated measure). Bonferroni test was used for pair-wise post-hoc analysis where there was a significant interaction in the data. A Welch’s corrected t-test was used where the difference in variance between groups was significantly different according to the Levene’s test. Data were expressed as mean ± s.e.m. and p values < 0.05 were considered statistically significant. Details of all statistics used are listed in Additional file 4: Table S1.

## Results

### Conditional deletion of 4EHP in CaMKIIα-positive excitatory neurons

**Fig. 1:**
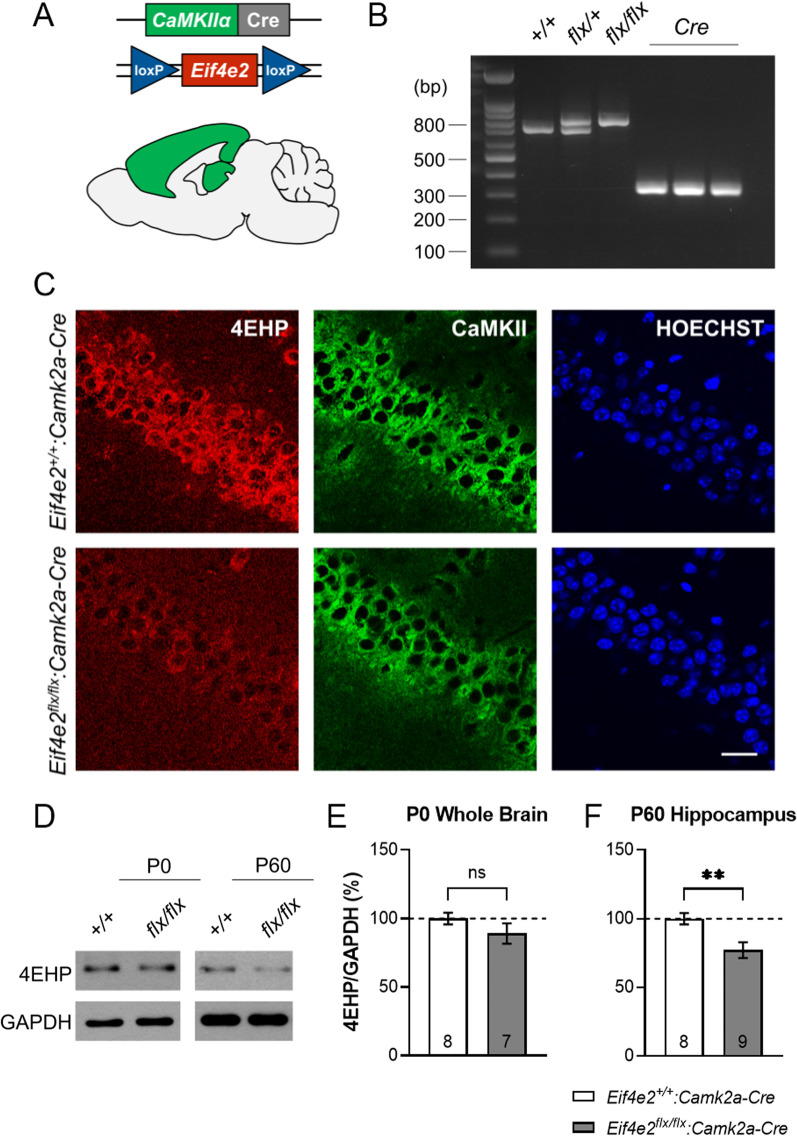
Conditional deletion of 4EHP in CaMKIIα-positive excitatory neurons. **A** Schematic depicting forebrain and hippocampal regions mainly (but not exclusively) targeted for Cre recombinase expression under the promoter of *Camk2a*. *Eif4e2* was flanked with loxP sites on both alleles. **B** Genotyping confirms WT or homozygous flx/flx mice with Cre recombinase. **C** Immunofluorescence analysis on the CA1 region of hippocampal coronal slices confirms specific deletion of 4EHP (red) in CaMKII-positive excitatory neurons (green). Hoechst-stained nuclei are in blue, scale bar represents 20 µm. **D** Western blot analysis further validates loss of 4EHP protein in the hippocampus of P60 mice (WT n = 8, cKO^exc^ n = 9) but not in P0 whole brain (WT n = 8, cKO^exc^ n = 7). **E**, **F** are quantifications of **D**. Data are presented as mean ± s.e.m. **p < 0.01; ns, not significant. P value calculated using an unpaired t-test. Sample size is located within the bar graph for each group

We previously generated and studied an excitatory neuron-specific 4EHP KO mouse model which displayed impaired sociability, hyperactivity, and synaptic plasticity dysfunction which is reminiscent of autism spectrum disorder (ASD) [12]. We used the *Emx1* promoter to drive Cre expression in excitatory neurons since EMX1 is expressed during embryonic development [25]. Deleting 4EHP early in development was chosen to examine developmental effects which are characteristic of ASD. Here, we sought to preclude potential developmental effects of 4EHP on learning and memory by driving Cre expression under the *Camk2a* promoter, which was originally reported to operate at P19 [21]. More recent work has indicated activity at P14 [26, 27]. To generate excitatory neuron-specific deletion of 4EHP, we crossed *Eif4e2*^*flx/flx*^ with mice expressing Cre recombinase under the *Camk2a* promoter (Fig. 1A). Genotyping confirmed homozygous floxed alleles and the presence of Cre (Fig. 1B). Immunofluorescence imaging of the CA1 hippocampus using antibodies against 4EHP and CaMKII revealed successful cell-type-specific KO of 4EHP directed in excitatory neurons (Fig. 1C). We further confirmed that deletion of 4EHP occurred at P60 (by 22.90 ± 7.28%, Fig. 1D, F) but not at P0 (Fig. 1 D and E) as determined by western blotting. The remaining 4EHP protein in the hippocampus at P60 is possibly due to relatively high levels of expression in other cell types such as glia and inhibitory neurons.

### 4EHP is dispensable for long-term memory formation

**Fig. 2:**
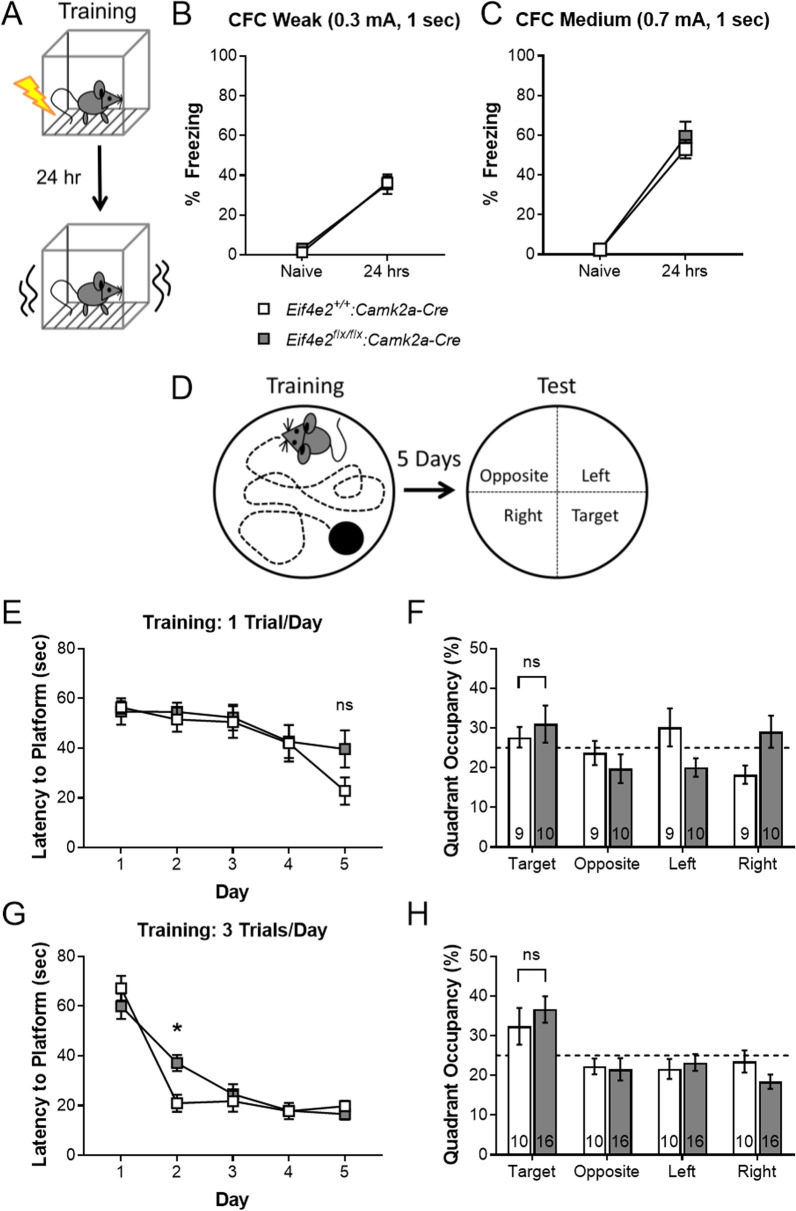
Long-term memory is normal in 4EHP-CaMKIIα KO mice. **A** Schematic depicting contextual fear conditioning (CFC) training and LTM test regime. **B** With weak CFC (0.3 mA, 1 s) training, 4EHP-cKO^exc^ mice (n = 10) show the same freezing behavior as WT (n = 8) 24 h after receiving a foot shock. **C** 24 h freezing behavior is not altered in 4EHP-cKO^exc^ mice (n = 8) compared to WT (n = 9) with a medium foot shock (0.7 mA, 1 s) training. **D** Training and testing paradigm for assessing memory in the MWM. Mice were trained to locate a hidden platform in a water maze using spatial cues for 5 days. On the 6^th^ day, the platform was removed, and mice were scored for time spent in each quadrant of the maze. **E** Learning curve graphed as latency to find platform on training days using a mild training protocol of 1 trial per day (WT n = 9, cKO^exc^ n = 10). **F** Percent time mice spent in each quadrant. Dashed line at 25% indicates no learning. **G** Learning curve graphed as latency to find platform on training days using a stronger training protocol of 3 trials per day (WT n = 10, cKO^exc^ n = 16). **H** Percent time mice spent in each quadrant. Dashed line at 25% indicates no learning. Data are presented as mean ± s.e.m. *p < 0.05; ns, not significant. P value calculated using 2-way ANOVA repeated measures with Bonferroni multiple comparisons test. Sample size is located within the bar graph for each group

Given the necessity of de novo protein synthesis in LTM formation, we first tested 4EHP-cKO^exc^ mice for long-term contextual fear memory (Fig. 2A). Mice were placed in a context with an electric grid floor and scored for naïve freezing prior to receiving either a weak (0.3 mA) foot shock for 1 s or a medium strength (0.7 mA) foot shock for 1 s. The weak shock was used to prevent a ceiling effect of freezing behavior and allow discrimination of possible memory enhancement in 4EHP-cKO^exc^ mice. LTM was assessed by quantifying freezing behavior 24 h following the foot shock. Under both conditions, LTM was intact in 4EHP-cKO^exc^ mice (Fig. 2B, C). Next, we subjected mice to the MWM spatial navigation learning and memory task (Fig. 2D). Mice were trained to locate a hidden platform over 5 days having been given a training protocol of either 1 trial per day (weak) or 3 trials per day (strong). With a weak training protocol, mice decreased the time needed to find the platform after 5 days (Fig. 2E), but did not show memory retention on the 6^th^ day test where the hidden platform was removed, and mouse quadrant occupancy was scored (Fig. 2F). Given a strong training protocol, 4EHP-cKO^exc^ mice had a significantly increased latency to locate the hidden platform on training day 2 by 16 s but were comparable to WT on days 3 to 5 (Fig. 2G). Both WT and 4EHP-cKO^exc^ mice showed a similar preference for the target quadrant during the test on the following day (Fig. 2H). Importantly, mice significantly decreased the time needed to find the platform during training (Fig. 2E, p = 0.0002; Fig. 2G , p< 0.0001; Additional file 4: Table S1). Together, these data indicate that 4EHP in excitatory neurons does not impact LTM.

### Spatial working memory requires 4EHP in excitatory neurons

**Fig. 3:**
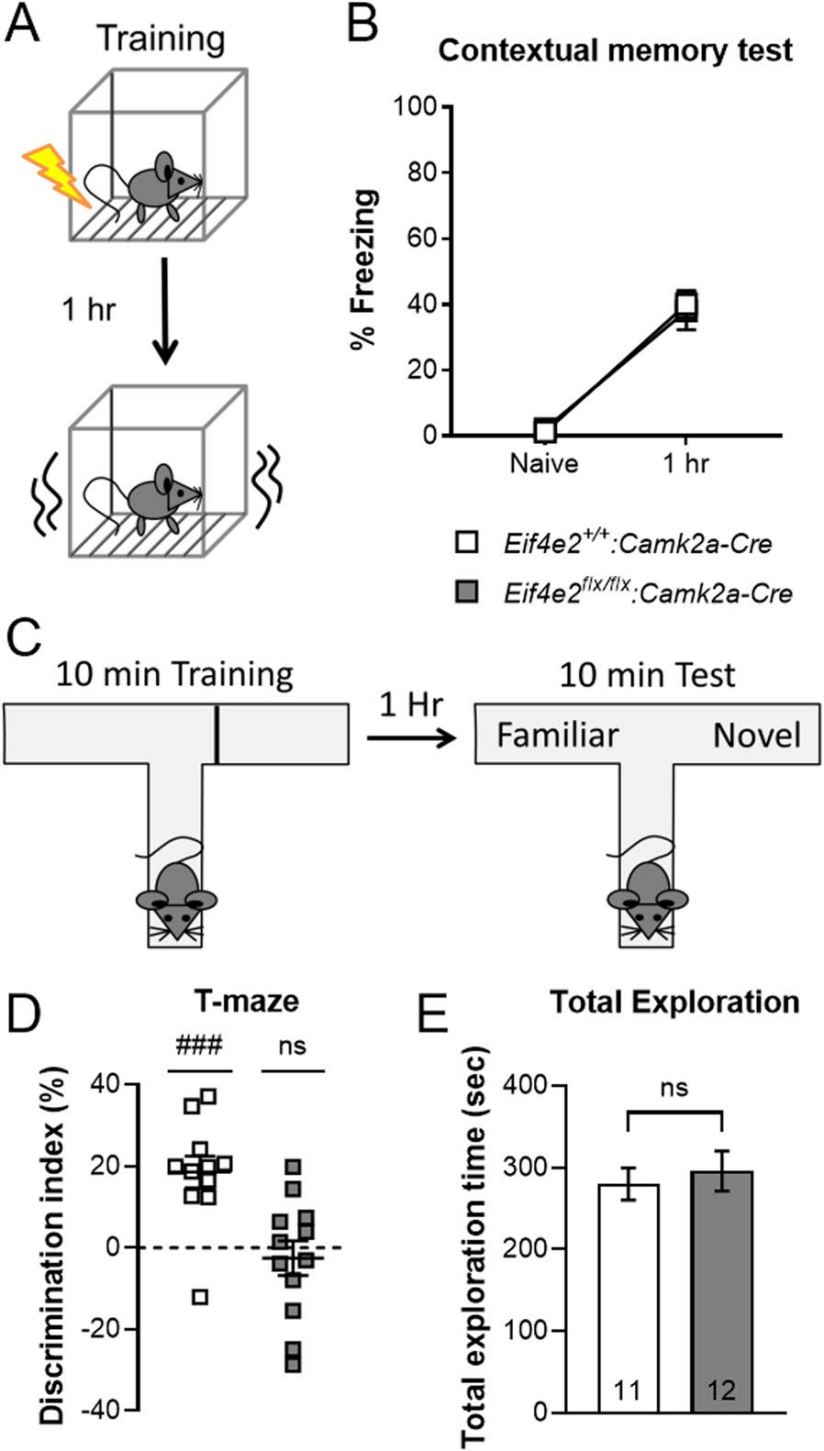
Working memory is impaired in 4EHP-CaMKIIα KO mice. **A** Schematic depicting contextual fear conditioning (CFC) training and short-term memory test regime. **B** Short-term contextual fear memory calculated by % freezing 1 h after receiving a mild foot shock (0.3 mA, 1 s) (WT n = 10, cKO^exc^ n = 9). **C** Schematic depicting T-maze spatial WM test. Mice were placed in a T-maze facing away from the junction (starting point) with one of either arm blocked for 10 min. After 1 h, mice were placed back in the maze with free access to both arms for 10 min of exploration. **D** T-maze memory is shown as a discrimination index for the novel vs. familiar arm where 0% means equal time spent in both arms (WT n = 11, cKO^exc^ n = 12). **E** Total exploration in either arm. Data are presented as mean ± s.e.m. ^###^p < 0.001; ns, not significant. P value calculated using one sample t-test or unpaired t-test. Sample size is located within the bar graph for each group

Working memory (WM), as opposed to LTM, is the short-term storage and manipulation of information for the guidance of decision-making behavior [28]. Deletion of the translational repressor protein 4E-BP2 leads to impairment of WM in mice [29]. This finding suggests that cap-dependent translation controls WM, but the mechanistic details are largely unknown. We tested 4EHP-cKO^exc^ and WT mice in a T-maze spatial WM task (Fig. 3C). Similar to 4E-BP2 KO mice, we observed robust WM impairment in 4EHP-cKO^exc^ mice compared to WT controls (Fig. 3D). Total exploratory behavior, which serves as a control for potential confounding effects of impaired exploratory behavior on memory, was not affected (Fig. 3E). The memory impairments were specific to spatial WM, as short-term (1 h, Fig. 3A) contextual fear memory was not impacted (Fig. 3B). We further validated these findings using an object recognition WM test (Additional file 1: Fig. S1A). Consistent with results from the T-maze, 4EHP-cKO^exc^ demonstrated impaired object recognition WM (Additional file 1: Fig. S1B) without changes in total exploration (Additional file 1: Fig. S1C). From these data, we conclude that 4EHP in excitatory neurons is necessary for intact spatial WM.

### Sustained inhibition of translation impairs working memory

**Fig. 4:**
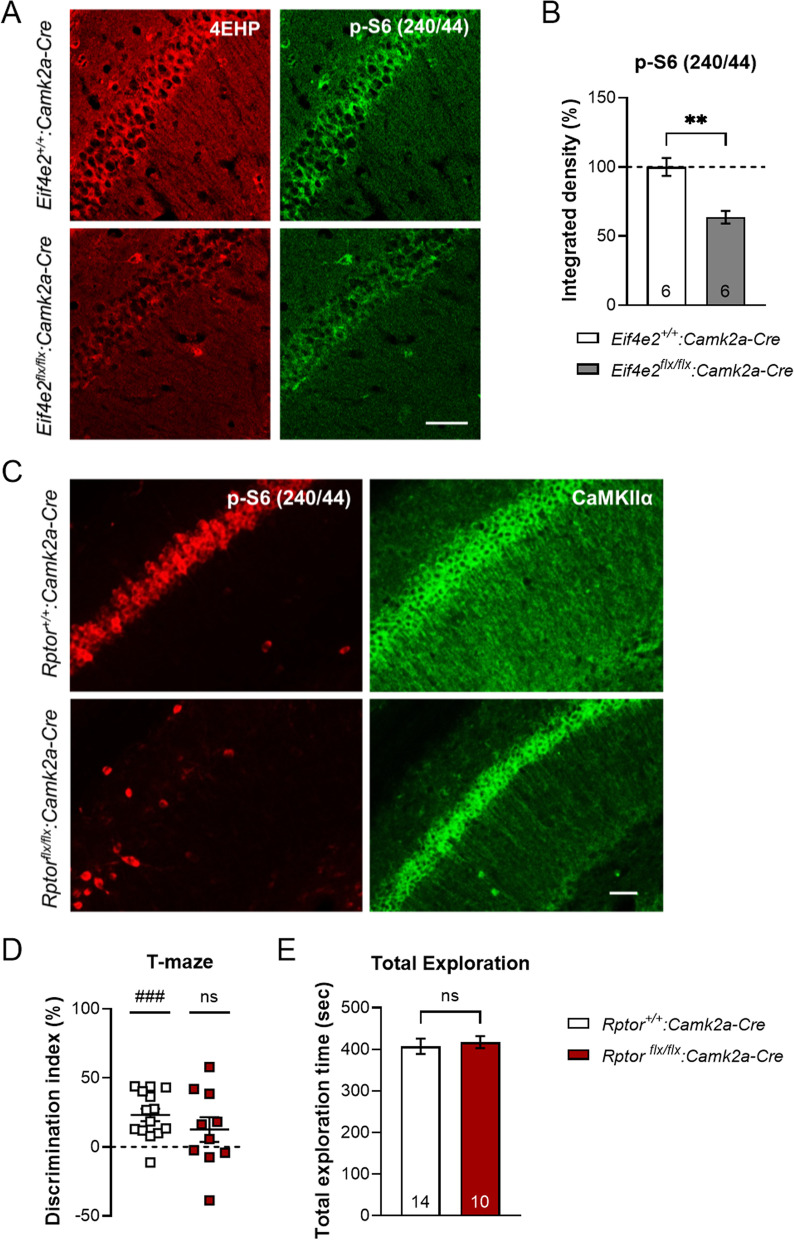
Working memory is impaired after sustained inhibition of protein synthesis. **A** Immunofluorescence analysis of p-S6 (Ser240/244) in 4EHP-cKO^exc^ vs. WT mouse hippocampus. **B** Quantification of p-S6 integrated density was performed on CA1 pyramidal neurons using image J. Scale bar represents 60 µm. **C** Immunofluorescence analysis of p-S6 (Ser240/244) in *Rptor*^*flx/flx*^*:Camk2a-Cre* mouse hippocampus compared to *Rptor*^+*/*+^*:Camk2a-Cre*. **D** Discrimination index for T-maze working memory in *Rptor*^*flx/flx*^*:Camk2a-Cre* (n = 14) compared to *Rptor*^+*/*+^*:Camk2a-Cre* (n = 10). **E** Total exploration time of both maze arms. Data are presented as mean ± s.e.m.; **p < 0.01; ns, not significant. P value calculated using unpaired t-test. ^###^p < 0.001, calculated using one sample t-test. Sample size is located within the bar graph for each group

We next sought to elucidate how loss of 4EHP disrupts WM based on its known function in mediating miRNA-induced translational silencing [8]. We previously performed an unbiased ribosome profiling study where the translational efficiency (TE) of mRNAs in mouse embryonic fibroblasts (MEFs) lacking 4EHP expression were determined compared to WT [9]. In this study, the ERK1/2 signalling pathway was negatively regulated by 4EHP deletion via translational upregulation of the ERK1/2 phosphatase *Dusp6* [9]. However, we did not previously observe changes in p-ERK1/2 in 4EHP KO brain [12]. Given that miRNAs have been shown to regulate the mTOR pathway activity in the brain [18], we investigated the activity of mTORC1 in the hippocampus of 4EHP-cKO^exc^ mice using immunofluorescence analysis. We observed a 36.40 ± 7.96% reduction in ribosomal protein S6 phosphorylation (which is a readout of mTOR activity) at serine position 240 and 244 [p-S6 (S240/44)] in 4EHP-cKO^exc^ excitatory neurons (Fig. 4A, B), consistent with reduced mTORC1 activity. Furthermore, mice lacking the defining component of the mTORC1 complex (Raptor) in excitatory neurons (*Rptor*^*flx/flx*^*:Camk2a-Cre*), which results in reduced p-S6 (S240/44) levels (Fig. 4C), have impaired WM (Fig. 4D), without changes in total exploratory behavior (Fig. 4E). Together, these data suggest that WM is impaired because of prolonged attenuation of mTORC1 via loss of 4EHP.

### Spatial working memory requires 4EHP and mTORC1 in inhibitory neurons

**Fig. 5:**
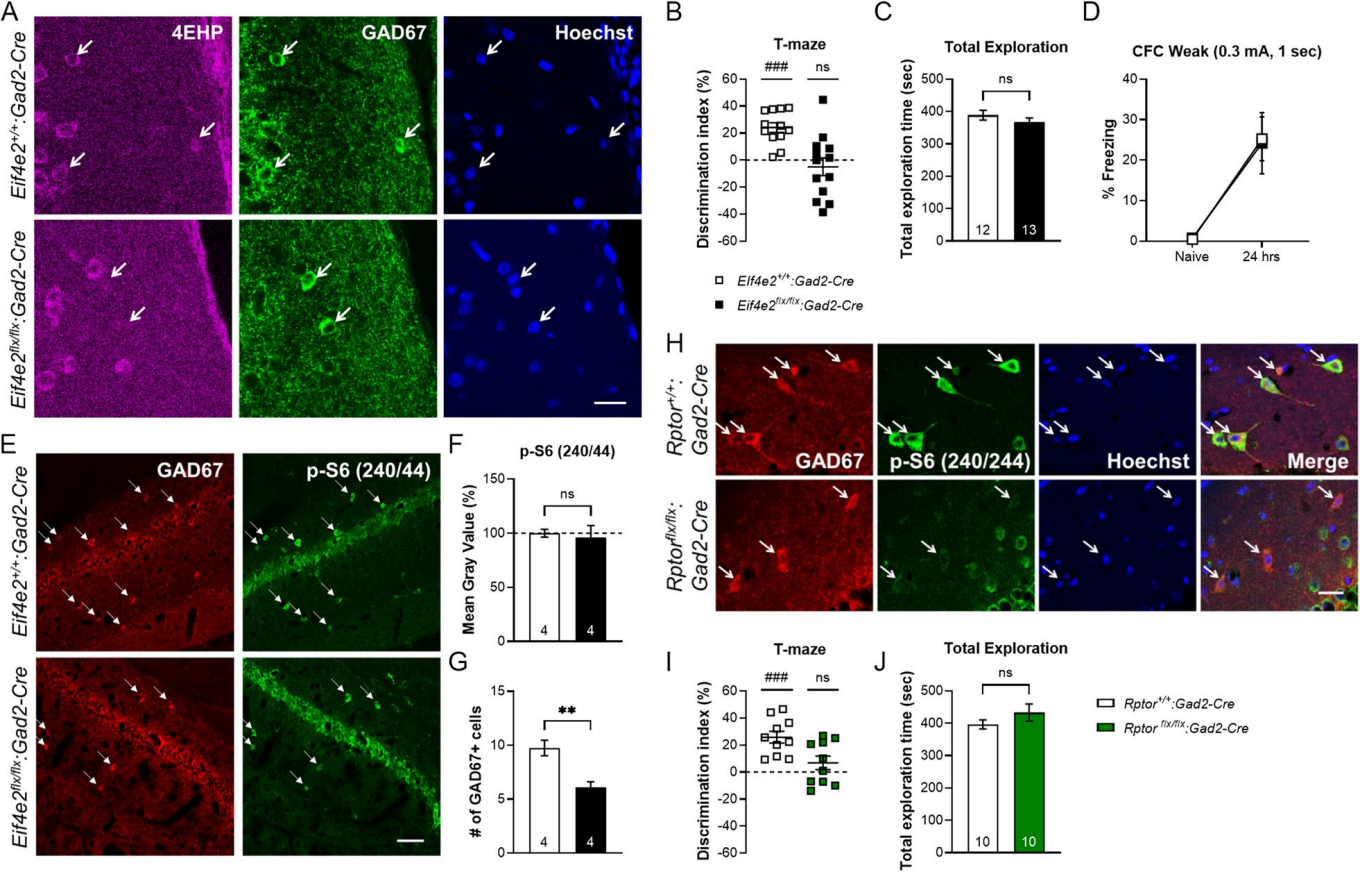
Inhibitory neurons mediate working memory via 4EHP and mTORC1. **A** Immunofluorescence analysis on prefrontal cortex coronal slices confirms specific deletion of 4EHP (purple) in GAD67-positive inhibitory neurons (green). Hoechst-stained nuclei are in blue, scale bar represents 20 µm. **B** T-maze memory is shown as a discrimination index for the novel vs. familiar arm where 0% means equal time spent in both arms (WT n = 12, cKO^inh^ n = 13). **C** Total exploration in either arm. **D** With weak CFC (0.3 mA, 1 s) training, 4EHP-cKO^inh^ mice (n = 9) show same freezing behavior as WT (n = 9) 24 h after receiving the foot shock. **E** Immunofluorescence analysis of p-S6 (Ser240/244) in 4EHP WT vs. cKO^inh^. GAD67-positive inhibitory neurons (identified with white arrows) are displayed in red and p-S6 in green. Scale bar represents 60 µm. **F** Quantification of Mean Gray Value, which is the Integrated Density normalized to area, revealed no difference in p-S6 fluorescence intensity between 4EHP WT (n = 4) and cKO^inh^ (n = 4). **G** The number of GAD67 cells were counted in 4EHP-cKO^inh^ (n = 4) vs. WT (n = 4). **H** Immunofluorescence analysis of p-S6 (Ser240/244) in *Rptor*^*flx/flx*^*:Gad2-Cre* mouse hippocampus compared to *Rptor*^+*/*+^*:Gad2-Cre*, scale bar represents 20 µm. **I** Discrimination index for T-maze working memory in *Rptor*^*flx/flx*^*:Gad2-Cre* (n = 10) compared to *Rptor*^+*/*+^*:Gad2-Cre* (n = 10). **J** Total exploration time of both maze arms. Data are presented as mean ± s.e.m. **p < 0.01; ns, not significant. P value calculated using an unpaired t-test. ^###^p < 0.001, calculated using one sample t-test. Sample size is located within the bar graph for each group

We next investigated WM in inhibitory neuron-specific 4EHP KO mice (4EHP-cKO^inh^). We first validated the deletion of 4EHP in GABAergic (GAD67-positive) neurons by immunofluorescence (Fig. 5A) in the prefrontal cortex where 4EHP is primarily expressed in neuronal cell types (Additional file 2: Fig. S2). Like 4EHP-cKO^exc^ mice, 4EHP-cKO^inh^ also demonstrate WM impairment (Fig. 5B) without changes in total exploratory behavior (Fig. 5C). Long-term contextual fear memory was not affected when 4EHP was deleted in inhibitory neurons (Fig. 5D). We explored the possibility that 4EHP regulates mTORC1 activity in inhibitory neurons by quantifying p-S6 in the GAD67-positive neurons. When normalized to the area of the cell, we did not detect a difference in p-S6 fluorescence signal in 4EHP-cKO^inh^ compared to WT (Fig. 5E, F). However, the total number of GAD67-positive cells was significantly reduced (37.82 ± 9.24%) in 4EHP-cKO^inh^ (Fig. 5G). Importantly, mTORC1 activity is still required for WM in inhibitory neurons as *Gad2*-directed deletion of Raptor (Fig. 5H) impaired WM (Fig. 5I) without affecting total exploratory behavior (Fig. 5J). We conclude that both 4EHP and mTORC1 in inhibitory neurons are required for proper WM.

## Discussion

Rapid de novo protein synthesis in neurons is required for forming long-term memories [30]. Temporally inhibiting eIF4E-dependent translation during consolidation prevents LTM formation [31]. Similarly, translational control via phosphorylation of eIF2α on Ser 51 acts as a critical gatekeeper of LTM as the non-phosphorylatable Ser51Ala mutant mice have a lower threshold for L-LTP and enhanced LTM [2, 3]. Given the necessity of de novo protein synthesis for LTMs and the emerging importance of microRNAs in synaptic plasticity and memory [11], we investigated the role of 4EHP in LTM. To this end, we subjected 4EHP conditional KO mice to a variety of LTM tests. First, using a contextual fear memory paradigm (CFC), we tested both 4EHP excitatory and inhibitory neuronal KO mice for memory 24 h post-training. In both models, memory retention was comparable to WT controls (Fig. 2B, C and Fig. 5D, respectively). We further assessed 4EHP-cKO^exc^ mice in the MWM where we did not observe any changes to LTM given either a mild or strong training regime (Fig. 2D–H). Given these results, we conclude that 4EHP is not required in excitatory or inhibitory neurons for LTM.

Earlier studies documented WM impairments in mice lacking mRNA translation-repressing proteins, such as 4E-BP2 [29] and PERK (an eIF2α kinase) [32, 33]. Here, we show that a third translational suppressor, 4EHP is required for WM in both excitatory and inhibitory neurons (Figs. 3D and 5B). Since deletion of 4EHP in either cell type abolishes WM, we conclude that the coordinated function of excitatory and inhibitory neurons mediates WM, as opposed to each cell type independently promoting WM.

We previously showed that mice lacking 4EHP in excitatory neurons defined by EMX1 exhibit impaired preference for social novelty in the 3-chamber sociability assay [12]. It can be argued that this impairment is mediated by a WM deficiency, rather than social interaction. However, 4EHP-EMX1 KO mice were also impaired in the direct/reciprocal social assay which does not depend on memory retention of a previous social encounter, unlike the 3-chamber social interaction test. Furthermore, 4EHP-cKO^inh^ mice, which have impaired WM, do not show ASD-like impairments, i.e., social deficits in the 3-chamber assay or exaggerated grooming behavior (Additional file 3: Fig. S3). This raises an intriguing possibility that 4EHP function is not only important for social behavior, but also for cognition in general. This is consistent with findings that executive functions, such as WM, are often impaired in patients with ASD [34–36]. Together these studies on 4EHP not only shed light on the mechanistic complexity mediating memory but may offer a potential therapeutic avenue for treating neurodevelopmental disorders such as ASD.

In attempts to understand how 4EHP mediates WM at the cellular and molecular level, we observed a reduction in p-S6 levels in 4EHP null excitatory neurons (Fig. 4A, B), consistent with attenuated activity of mTORC1 signalling. One possible explanation for this finding is that 4EHP represses the translation of mRNAs which encode for proteins that control this signalling pathway. This type of regulation was previously observed in an unbiased ribosome profiling study where the translational efficiency (TE) of mRNAs in mouse embryonic fibroblasts (MEFs) lacking 4EHP expression were determined compared to WT [9]. In this study, TE of the ERK1/2 phosphatase *Dusp6* was significantly upregulated in 4EHP KO MEFs which resulted in reduced p-ERK1/2 levels, impairments in cell proliferation, and increased apoptosis [9]. In our previous study, we did not observe changes in p-ERK1/2 in 4EHP KO brain [12]. Another possibility is that reduced mTORC1 activity in cells lacking 4EHP may be a compensatory mechanism to maintain proteostasis. This would be consistent with our previous results showing that global rates of de novo protein synthesis are not changed in the absence of 4EHP in the brain [12]. However, reduction in mTORC1 activity itself can drive WM impairments, as deletion of Raptor alone engendered impaired WM (Fig. 4C, D). This suggests that mTORC1 activity regulates the expression of critical factors involved in WM. In this case, the WM impairments in 4EHP-cKO^exc^ mice are likely a direct consequence of reduced mTORC1 activity by 4EHP-mediated translational control of factors that regulate the mTORC1 pathway.

We further assessed whether mTORC1 activity in 4EHP-cKO^inh^ mice is dysregulated. We did not observe a difference in p-S6 in GAD67-positive neurons in 4EHP-cKO^inh^ compared to controls (Fig. 5E, F). Instead, the total number of GAD67 cells was reduced in 4EHP-cKO^inh^ mice, indicating a developmental abnormality; this is possibly due to the early expression of Cre in *Gad2-Cre* mice [22]. These results are consistent with the known role of 4EHP in proper mammalian development, as full-body 4EHP KO mice are born underdeveloped and fail to survive [20]. This phenotype may be underlying the observed WM impairment, but further research is required to establish a causal relationship. Deletion of Raptor in inhibitory neurons was sufficient to impair WM (Fig. 5H, I). Together, our results suggest that 4EHP and sustained mTORC1 activity in excitatory or inhibitory neurons is necessary to support WM. Future research is required to determine whether mTORC1 is directly modulated by 4EHP and miRNA-mediated translational control to mediate WM. These mechanistic studies will ideally be carried out in multiple brain regions which are important for WM, such as the hippocampus [37] and prefrontal cortex [38].

## Limitations

It is necessary to understand how 4EHP mechanistically regulates mTORC1 activity. The hypothesis that 4EHP controls the translation of kinases and/or phosphatases in the mTORC1 pathway could be studied using an unbiased mRNA translation analysis approach, such as Translating Ribosome Affinity Purification (TRAP) [39] or RiboTag, according to our previous work [3]. In these methods, a tagged ribosomal protein is expressed through an adeno-associated virus (AAV) under Cre recombinase control. After purifying and sequencing ribosome-bound mRNAs, we can evaluate ribosome occupancy on mRNAs as a measure of active translation across genotypes [40]. This would enable the analysis of the mRNAs in cell types that are translationally regulated by 4EHP.

## Supplementary Information

**Additional file 1: Figure S1.** Object recognition working memory. **A** Mice were assessed for working memory using a novel object recognition task (NOR). **B** NOR memory is shown as a discrimination index for the novel vs. familiar object where 0% means equal time exploring both objects (WT n = 7, cKO^inh^ n = 10). **C** Total exploration of objects. Data are presented as mean ± s.e.m. ^#^p < 0.05, calculated using one sample t-test. Sample size is located within the bar graph for each group.

**Additional file 2: Figure S2.** 4EHP expression pattern in the prefrontal cortex. Analysis of cell-type-specific expression of 4EHP by colocalization with **A** Empty Spiracles Homeobox 1 (EMX1, defining excitatory neurons), **B** parvalbumin (PV, defining a subset of inhibitory neurons), **C** somatostatin (SST, defining another subset of inhibitory neurons), and **D** laminin (LAMA1, defining endothelial cells) in the prefrontal cortex of WT mice. 4EHP expression is colored in red, the cell type marker in green, and Hoechst-stained nucleus in blue. Arrows indicate a positive signal for the cell type maker. Scale bar represents 20 µm.

**Additional file 3: Figure S3.** Inhibitory interneuron-specific deletion of 4EHP does not affect ASD-like behaviors. Assessment of ASD-like behaviors in mice lacking 4EHP expression specifically in inhibitory neurons defined by *Gad2*. **A** Total time spent grooming. **B** Number of grooming bouts. **C** Sniffing time between either an empty cage (E) or a cage containing a stranger mouse (S1). **D** Sniffing time between either the previously encountered stranger mouse (S1) or a novel stranger mouse (S2). Data are presented as mean ± s.e.m.; **p < 0.01, ***p < 0.001, ns., not significant; calculated by unpaired t-test or 2-way ANOVA with Bonferroni multiple comparisons test. Sample size is located within bar graphs.

**Additional file 4: Table S1.** Summary of statistics

## Data Availability

The data and analysis for the work presented in the current study are available from the corresponding author upon request.

## Abbreviations

eIF: Eukaryotic initiation factor
4EHP: Eukaryotic initiation factor 4E homologous protein
CKO^exc^: : Conditional knockout in *Camk2a*-expressing cells
CKO^inh^: : Conditional knockout in *Gad2*-expressing cells
LTM: Long-term memory
MiRISC: : MiRNA-induced silencing complex
4E-T: EIF4E transporter protein
P: Postnatal day
RT: Room temperature
E: Embryonic day
RIPA: Radioimmunoprecipitation assay
BSA: Bovine serum albumin
TBST: Tris-Buffered Saline with Tween 20
HRP: Horseradish peroxidase
MGV: Mean gray value
MWM: Morris water maze
ASD: Autism spectrum disorder
WM: Working memory
MEF: Mouse embryonic fibroblast
CFC: Contextual fear memory
TRAP: Translating ribosome affinity purification
