# Supplementary figures and images for: Cell-type-specific translational control of spatial working memory by the cap-binding protein 4EHP
Source: Mol Brain. 2023 Jan 18;16:9. doi: 10.1186/s13041-023-00995-2 (PMC9847188; doi:10.1186/s13041-023-00995-2)

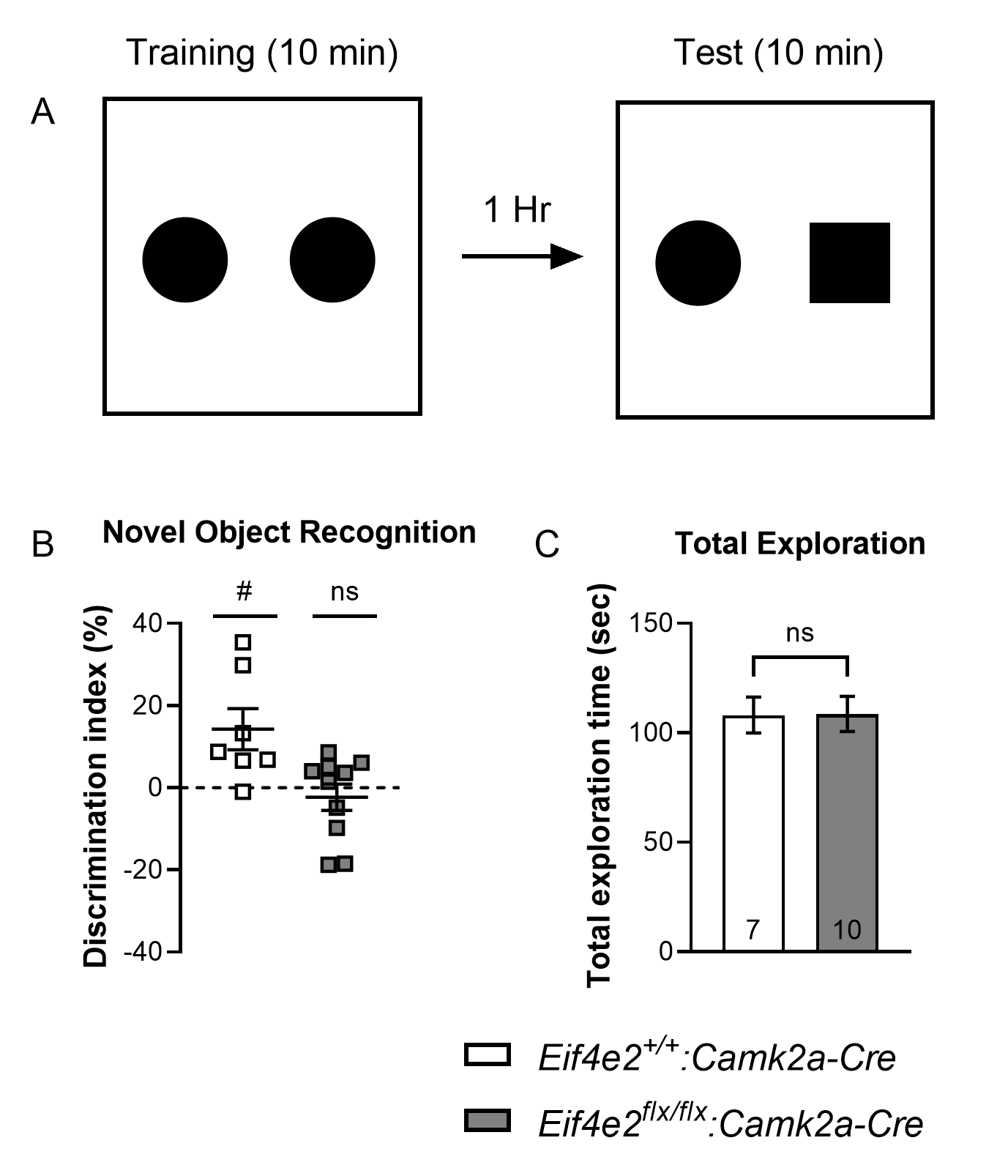

Supplement: Supplementary file 1 — Additional file 1: Figure S1. Object recognition working memory. A Mice were assessed for working memory using a novel object recognition task (NOR). B NOR memory is shown as a discrimination index for the novel vs. familiar object where 0% means equal time exploring both objects (WT n = 7, cKOinh n = 10). C Total exploration of objects. Data are presented as mean ± s.e.m. #p < 0.05, calculated using one sample t-test. Sample size is located within the bar graph for each group. [file 13041_2023_995_MOESM1_ESM.tif]

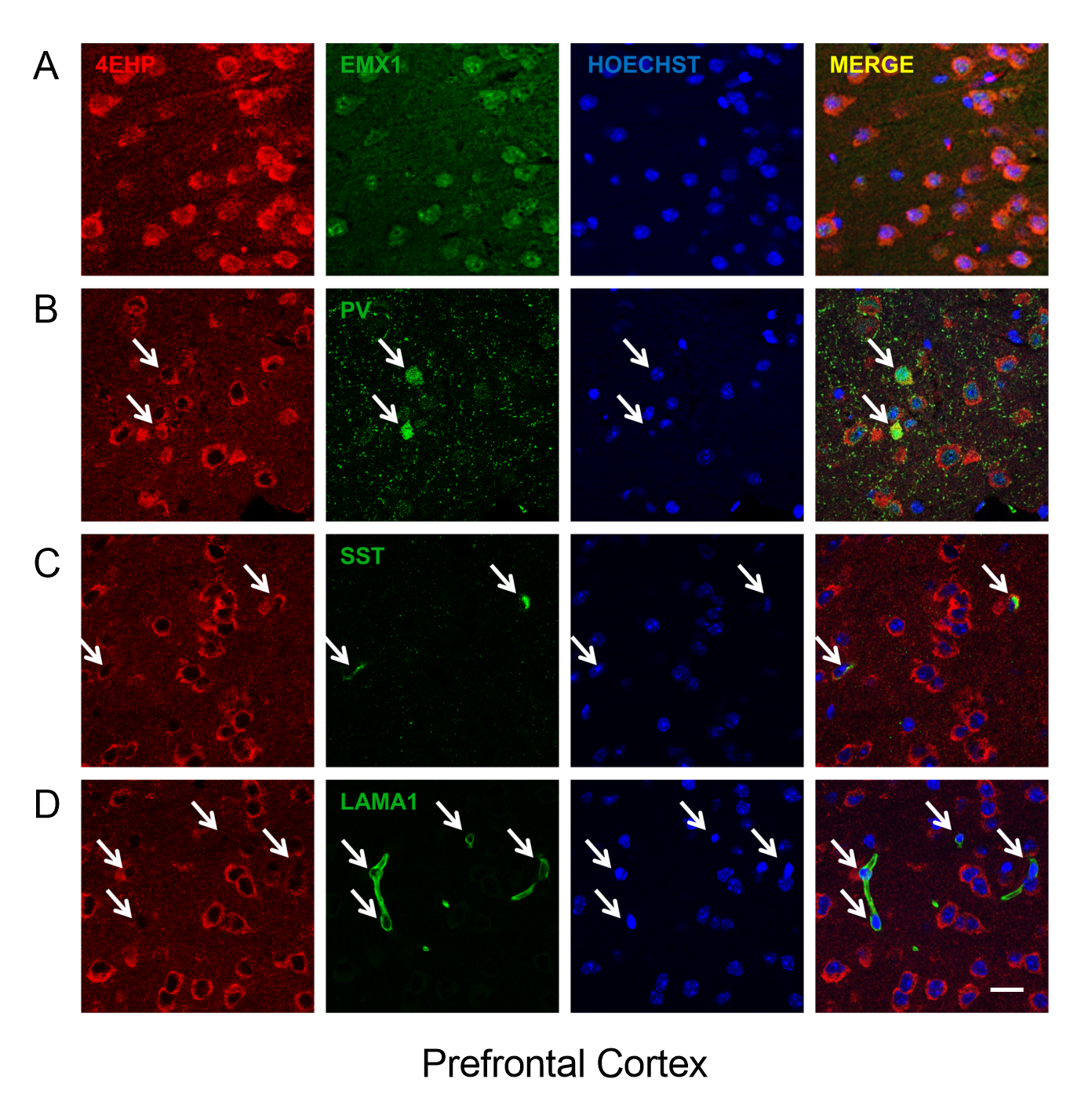

Supplement: Supplementary file 2 — Additional file 2: Figure S2. 4EHP expression pattern in the prefrontal cortex. Analysis of cell-type-specific expression of 4EHP by colocalization with A Empty Spiracles Homeobox 1 (EMX1, defining excitatory neurons), B parvalbumin (PV, defining a subset of inhibitory neurons), C somatostatin (SST, defining another subset of inhibitory neurons), and D laminin (LAMA1, defining endothelial cells) in the prefrontal cortex of WT mice. 4EHP expression is colored in red, the cell type marker in green, and Hoechst-stained nucleus in blue. Arrows indicate a positive signal for the cell type maker. Scale bar represents 20 µm. [file 13041_2023_995_MOESM2_ESM.tif]

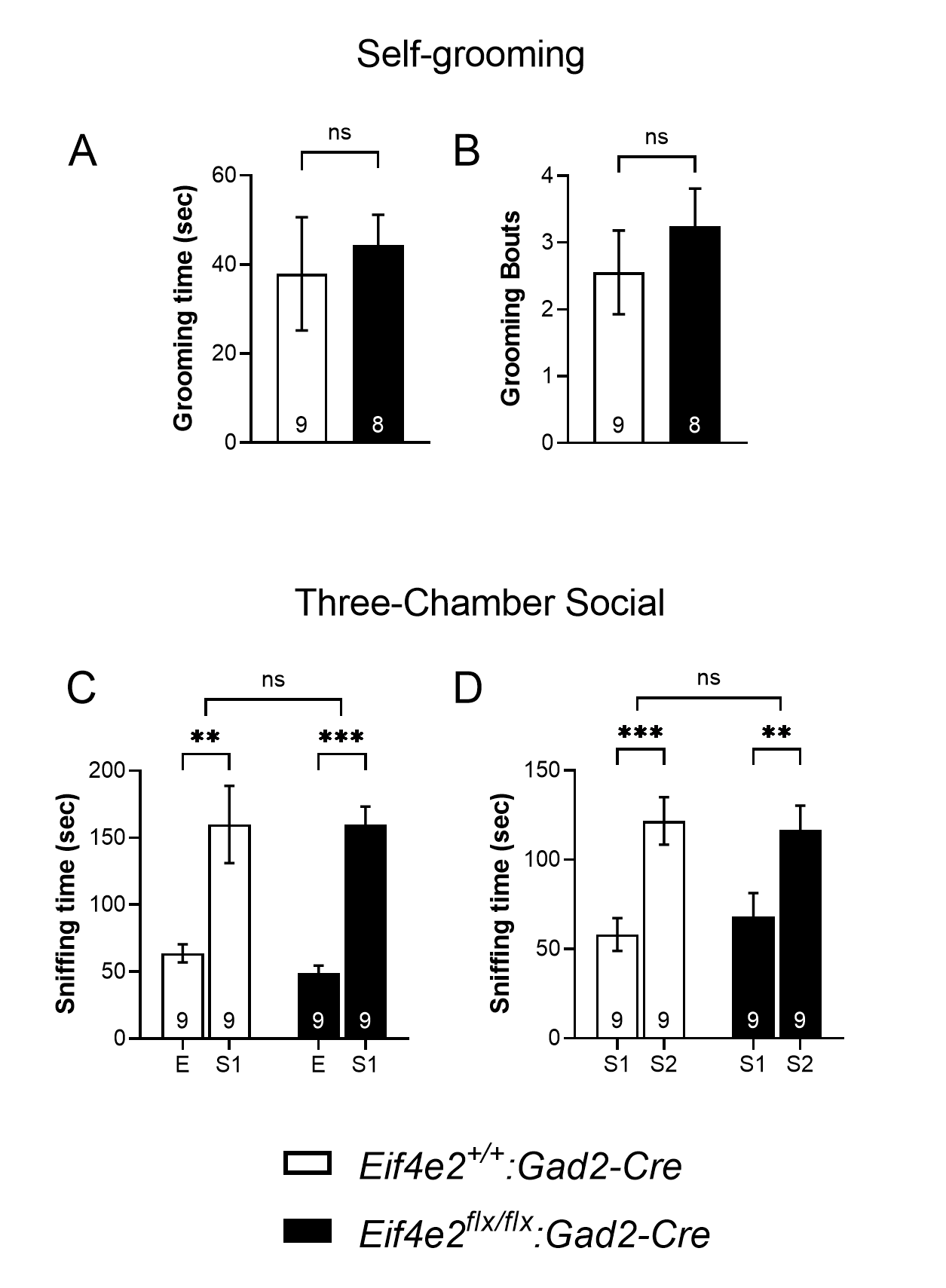

Supplement: Supplementary file 3 — Additional file 3: Figure S3. Inhibitory interneuron-specific deletion of 4EHP does not affect ASD-like behaviors. Assessment of ASD-like behaviors in mice lacking 4EHP expression specifically in inhibitory neurons defined by Gad2. A Total time spent grooming. B Number of grooming bouts. C Sniffing time between either an empty cage (E) or a cage containing a stranger mouse (S1). D Sniffing time between either the previously encountered stranger mouse (S1) or a novel stranger mouse (S2). Data are presented as mean ± s.e.m.; **p < 0.01, ***p < 0.001, ns., not significant; calculated by unpaired t-test or 2-way ANOVA with Bonferroni multiple comparisons test. Sample size is located within bar graphs. [file 13041_2023_995_MOESM3_ESM.tif]
